# Hypertension management profiles in Chinese adults aged 60 years and older

**DOI:** 10.3389/fpubh.2026.1807538

**Published:** 2026-05-19

**Authors:** Jiayi Xia, Yeping Chen, Dongmei Pu, Xiaoyan Jiang, Yan Liu, Song Pei

**Affiliations:** 1 Clinical Research Center, The Second Rehabilitation Hospital of Shanghai, Shanghai, China; 2 Administrative Department, Songnan Town Community Health Service Centre, Baoshan District, Shanghai, China; 3 Department of Rehabilitation Medicine, Renhe Hospital, Baoshan District, Shanghai, China

**Keywords:** aged, cluster analysis, home blood pressure monitoring, hypertension, self-management

## Abstract

**Background:**

Hypertension control in older adults depends on sustained self-management, yet education strategies often overlook heterogeneity in barriers and capacities.

**Objective:**

To examine hypertension-related knowledge, behaviors, and educational needs in Chinese adults aged 60 years and older, identify determinants of regular blood pressure (BP) monitoring, and derive management profiles for targeted intervention.

**Methods:**

A cross-sectional questionnaire survey was conducted among Chinese adults aged 60 years and older recruited from community-based and outpatient clinical settings in Shanghai (*n* = 316). Descriptive analyses summarized knowledge, behaviors, barriers, and preferred formats. Multivariable logistic regression identified factors associated with regular BP monitoring. K-means clustering and principal component analysis were used to define management profiles.

**Results:**

While 76.3% knew the ideal BP range, only 53.2% reported regular monitoring, despite 75.3% owning a home device. Older age predicted lower monitoring likelihood (OR = 0.944 per year, 95% CI 0.901–0.988, *p* = 0.013). Absence of a home monitor (OR = 0.113, *p* < 0.001) and lack of knowledge of standardized measurement procedures (OR = 0.212, *p* < 0.001) were strong negative predictors, whereas conceptual BP knowledge was not. Top education priorities were low-salt diet (69.3%) and older-adult-appropriate exercise (68.0%). Major barriers included information overload (64.6%) and technical terminology (56.6%). Face-to-face consultation was most preferred (81.3%). Five profiles showed marked heterogeneity (all P_FDR < 0.001). The High-knowledge/regular-monitoring profile had the lowest non-regular monitoring (29.2%). The Low-monitoring/low-resource profile had the highest non-regular monitoring (83.6%) and knowledge deficit (27.3%). The Medication-problem profile showed the most adherence problems (37.0%).

**Conclusion:**

Among Chinese adults aged 60 years and older, regular BP monitoring appears to be influenced more by operational capability than by conceptual awareness. Profile-guided, barrier-aware education may enhance the precision and scalability of hypertension self-management in similar community-based and outpatient care settings.

**Clinical trial registration:**

https://www.chictr.org.cn/, ChiCTR2600116499.

## 1 Introduction

Hypertension remains one of the most prevalent and modifiable contributors to cardiovascular morbidity and mortality worldwide, with its burden rising in parallel with population aging (1). In China, hypertension prevalence increases markedly with age, and recent evidence indicates that the burden is particularly concentrated in older adults, among whom prevalence remains high (47%) and hypertension is associated with adverse health outcomes, including unfavorable cognitive trajectories (2–4). In addition, multimorbidity is common in older adults with hypertension and may complicate management and reduce BP control rates (5). Polypharmacy and functional decline, including frailty and reduced physiological reserve, may further amplify the risk of uncontrolled BP and adverse outcomes such as stroke, myocardial infarction, and heart failure (6). Hypertension in older adults has also been associated with cognitive impairment and functional disability, which may undermine effective self-care (7). In this population, effective hypertension management increasingly depends not only on access to medications and clinical follow-up, but also on sustained self-management behaviors, including regular monitoring of BP, adherence to treatment, and timely response to marked BP elevation detected during monitoring or warning signs suggestive of acute cardiovascular or cerebrovascular complications (8).

Hypertension self-management includes several interrelated components associated with improved BP control and reduced complications in older adults (9). Accurate BP measurement is foundational, because home BP monitoring and other out-of-office measures can help identify white-coat and masked hypertension that may be missed by sporadic office readings, thereby supporting more timely treatment adjustment and improved control (10, 11). Medication adherence is equally critical, yet adherence among older adults with hypertension remains inconsistent and frequently suboptimal across published studies, with a recent systematic review highlighting persistent barriers related to regimen complexity, financial status, health beliefs, and health literacy (12). Regular clinical follow-up may support risk stratification and regimen optimization, but attendance may be influenced by competing priorities, transportation barriers, and limited health literacy (13, 14). Lifestyle practices—particularly dietary sodium reduction, regular physical activity, and avoidance of smoking and excessive alcohol consumption—are consistently recommended by hypertension guidelines and have been shown to yield clinically meaningful reductions in BP and cardiovascular risk, especially in older adults (15, 16).

Despite this framework, effective self-management in real-world settings may be limited by how health information is delivered and used by older adults (17). Older adults are increasingly exposed to health information through online media, mobile messaging apps, and community channels, but this broader access also introduces variability in credibility, clarity, and relevance (18). The information environment may be fragmented, inconsistent, or overly technical, making reliable and actionable information harder to identify (19). Although these challenges are not unique to older adults, they may be especially salient in this population because age-related sensory and cognitive changes and variable digital health literacy can make it more difficult to identify, evaluate, and apply health information effectively (17, 20). Consequently, even when awareness of certain risk factors appears high, this awareness may not necessarily translate into stable self-management behaviors, particularly when educational resources are fragmented, difficult to verify, or insufficiently adapted to the needs of older users (18).

Another limitation of current evidence is that hypertension education is often designed from a provider-centered perspective and evaluated using broad outcomes, with insufficient attention to the individualized needs of older adults (21). Older populations are heterogeneous: knowledge deficits, access to home BP monitors, medication-related concerns, and preferred learning formats may vary across individuals and form distinct management profiles (22, 23). Without a clearer needs-based portrait, educational interventions may remain generic, poorly aligned with patient priorities, and difficult to scale effectively (24). A data-driven characterization of education needs and behavioral profiles may therefore help inform targeted messaging, channel selection, and resource allocation, potentially improving the translation of education into practical self-management behaviors (22).

To address these gaps, we conducted a cross-sectional survey among older adults to characterize hypertension-related knowledge, self-management behaviors, educational needs, barriers to information access, and preferred formats for receiving health education. The study was designed with three linked objectives: first, to provide a descriptive overview of hypertension-related knowledge, behaviors, and education needs; second, to examine factors associated with regular BP monitoring as a key actionable self-management behavior using multivariable logistic regression; and third, to identify broader management profiles through unsupervised clustering. By integrating descriptive epidemiology, behavior-focused modeling, and data-driven profile identification, this study aims to provide actionable evidence for designing older adult friendly, precision-oriented health education strategies that improve the usability and uptake of hypertension self-management recommendations in routine care.

## 2 Methods

### 2.1 Study design and setting

This study employed a cross-sectional observational design with both descriptive and analytical components, using a structured questionnaire survey to characterize hypertension-related knowledge, self-management behaviors, and health education needs among older adults. This design was chosen because the study aimed to describe the current distribution of these characteristics, examine factors associated with regular BP monitoring at a single time point, and identify broader management profiles in a real-world care context. Data were collected from January 1, 2026 to January 31, 2026 in communities surrounding the Second Rehabilitation Hospital of Shanghai, using community-based survey activities and outpatient clinical settings as recruitment sites. A convenience sampling strategy was adopted. Potential participants were approached on site by trained investigators, informed about the purpose of the study, and invited to participate voluntarily. All variables were obtained at a single time point to reflect the contemporaneous status of hypertension management practices and informational needs in the target population. The study protocol was registered with the Chinese Clinical Trial Registry (Registration No. ChiCTR2600116499) and approved by the institutional ethics committee of Shanghai Second Rehabilitation Hospital (Ethics Approval No. 2025-32-01).

### 2.2 Participants and sample size estimation

Eligible participants were Chinese adults aged 60 years and older who were approached during the study period in the participating community-based or outpatient settings, were able to understand the questionnaire, and provided written informed consent. Respondents were eligible individuals who completed the questionnaire. Respondents were excluded if they were aged <60 years, had missing key demographic information (e.g., age or sex), or if their questionnaires were deemed invalid after quality control, including substantial logical inconsistencies or incomplete core items required for primary analyses. All data were anonymized prior to analysis to ensure confidentiality.

To provide context for sample adequacy, the required sample size was estimated *post hoc* using the standard formula for cross-sectional surveys:

$$n=Z_{1-\alpha /2}^{2}\times p(1-p)/d^{2}$$

where Z_1 − *α*/2_ = 1.96 for a 95% confidence level, *p* was conservatively set at 0.50, and *d* represented the allowable error. Using an allowable error of 0.06, the minimum required sample size was approximately 267. The final analytical sample of 316 participants was therefore considered adequate for the descriptive, regression, and exploratory profile-based analyses conducted in this study.

### 2.3 Questionnaire development, content, and variables

A structured, study-specific questionnaire was administered in this study. The instrument was developed based on the study objectives, relevant literature on hypertension self-management and health education, and practical considerations related to BP monitoring, medication use, and health information needs in older adults. To enhance content validity, the draft questionnaire was reviewed by five experts from the fields of cardiovascular medicine, rehabilitation, nursing, epidemiology, and health management. Prior to formal administration, the questionnaire was pilot-tested in 12 older adults to assess comprehensibility, wording clarity, and feasibility, thereby supporting its face validity and practical usability. Minor wording adjustments were made accordingly. The complete questionnaire is provided in Appendix 1.

The questionnaire collected information on sociodemographic characteristics, anthropometric measures, and BP–related information. Sex was recorded as a binary categorical variable (male/female), age was recorded as a continuous variable in years, and education was recorded as a categorical variable with five levels: primary school or below, junior high school, senior high school/secondary technical school, junior college, and bachelor’s degree or above. Anthropometric measures included height and weight, and BP–related information included participants’ self-reported recent BP fluctuation ranges, recorded as lower and upper values for systolic and diastolic BP.

Hypertension-related knowledge was assessed by four items covering awareness of the ideal/normal BP range, awareness of standardized BP measurement rules, and awareness of the health harms associated with smoking and alcohol consumption. A simple hypertension knowledge score was calculated by summing these four items, with each correct/affirmative response coded as 1 and each incorrect/negative response coded as 0, yielding a total score ranging from 0 to 4. Because the score range was narrow and the distribution was non-normal, participants were classified into low knowledge level and high knowledge level groups using the median split method for the main analyses. The median knowledge score was 3; accordingly, scores of 0–2 were classified as low knowledge level, whereas scores of 3–4 were classified as high knowledge level.

Multi-choice items were coded as binary variables (selected = 1, not selected = 0). Summary counts (e.g., the number of educational needs or barriers selected) were generated as composite indicators for subsequent analyses. Body mass index (BMI) was calculated as weight (kg) divided by height squared (m^2^).

### 2.4 Outcomes

The primary outcome was regular BP monitoring, defined as a binary variable indicating whether participants reported measuring their BP on a regular basis. This outcome was selected because it represents a central, observable, and actionable component of hypertension self-management in older adults. In addition, regular BP monitoring serves as a practical behavioral bridge between knowledge, resource availability, and daily disease management, making it particularly suitable for explanatory modeling. Compared with broader composite self-management constructs, regular BP monitoring is directly relevant to out-of-office BP control, timely treatment adjustment, and health education implementation.

Secondary outcomes included hypertension-related knowledge indicators (awareness of the ideal/normal BP range, awareness of standardized BP measurement rules, and awareness of the harms of smoking and alcohol consumption), management resources and behaviors (ownership of a home BP monitor, use of antihypertensive medications, and follow-up behavior), and lifestyle-related variables (salt consumption habits, exercise duration, years of smoking, and years of alcohol consumption). Additional secondary outcomes captured medication-related behavior problems, including the presence and patterns of reported non-adherence behaviors, as well as health education needs, assessed by the selection rates of priority educational topics, perceived barriers, and preferred education formats. Furthermore, profile-level outcomes derived from unsupervised clustering were used to characterize heterogeneity in hypertension management and educational needs across subgroups.

### 2.5 Statistical analysis

Data management and statistical computations were performed using MATLAB R2020a (MathWorks Inc., Natick, MA, USA). A two-sided *p* < 0.05 was considered statistically significant. Statistical analyses included two main components: (1) descriptive statistical analysis, used to summarize participant characteristics, hypertension-related knowledge, self-management behaviors, and health education needs; and (2) inferential statistical analysis, including multivariable logistic regression, unsupervised k-means clustering, and between-cluster comparisons.

#### 2.5.1 Data preprocessing and descriptive statistics

Continuous variables were first assessed for normality using the Lilliefors test. Normally distributed data were presented as mean ± standard deviation (SD), while non-normally distributed variables (e.g., age, BMI, and BP metrics) were expressed as median and interquartile range (IQR). Categorical variables were summarized as absolute frequencies (n) and percentages (%). Participants aged below 60 years were excluded to ensure the cohort focused on older adults.

#### 2.5.2 Multivariable logistic regression

To identify independent factors associated with regular BP monitoring behavior, a multivariable logistic regression model was constructed. The model incorporated potential predictors including demographic characteristics (sex, age, and education), availability of home BP monitors, and disease-related knowledge (awareness of ideal BP and measurement rules). Age was entered as a continuous variable, whereas sex and education were entered as categorical variables. Education was modeled with primary school or below as the reference category. Odds ratios (ORs) and corresponding 95% confidence intervals (CIs) were calculated to evaluate the strength and direction of these associations. This model was intentionally focused on monitoring-related proximal determinants and was not intended to capture the full behavioral and psychosocial complexity of hypertension self-management.

#### 2.5.3 Unsupervised K-means cluster analysis

An unsupervised K-means clustering algorithm was employed to identify distinct management profiles of hypertension self-management and health needs among the participants. In contrast to the behavior-specific regression model, clustering was used to characterize multidimensional heterogeneity in hypertension management by integrating knowledge, behavior, and educational-need variables at the profile level. Feature Engineering: A multidimensional feature set was constructed, including demographic profiles, knowledge scores, medication-related issues, and categorical health education needs. All features were standardized via Z-score transformation prior to clustering to mitigate scaling bias.

Optimal cluster determination: The optimal number of clusters (K) was determined by evaluating the mean silhouette coefficient, which measures the cohesion and separation of the resulting groups.

Dimensionality reduction: Principal Component Analysis (PCA) was utilized to project the high-dimensional feature space into two dimensions for visualization and to validate the distinctness of the identified clusters.

Cluster labels were assigned *post hoc* for descriptive interpretability, based on the dominant distinguishing features of each group across key knowledge, behavior, and resource-related indicators, and should be interpreted as descriptive profile names rather than fixed latent constructs.

#### 2.5.4 Inferential statistics for cluster comparison

Inter-cluster differences were evaluated based on data distribution. One-way Analysis of Variance (ANOVA) with Tukey–Kramer post-hoc tests was applied for normally distributed continuous variables with homogeneous variance (confirmed by Levene’s test). For non-normally distributed data or those with heterogeneous variance, the Kruskal-Wallis H test was performed. Categorical variables were analyzed using the Chi-square (\chi^2) test, with effect sizes estimated using Cramer’s V. To minimize Type I errors in multiple comparisons, P -values for key factor differences were adjusted using the Benjamini-Hochberg False Discovery Rate (FDR) procedure.

## 3 Results

### 3.1 Baseline characteristics

**Figure 1:**
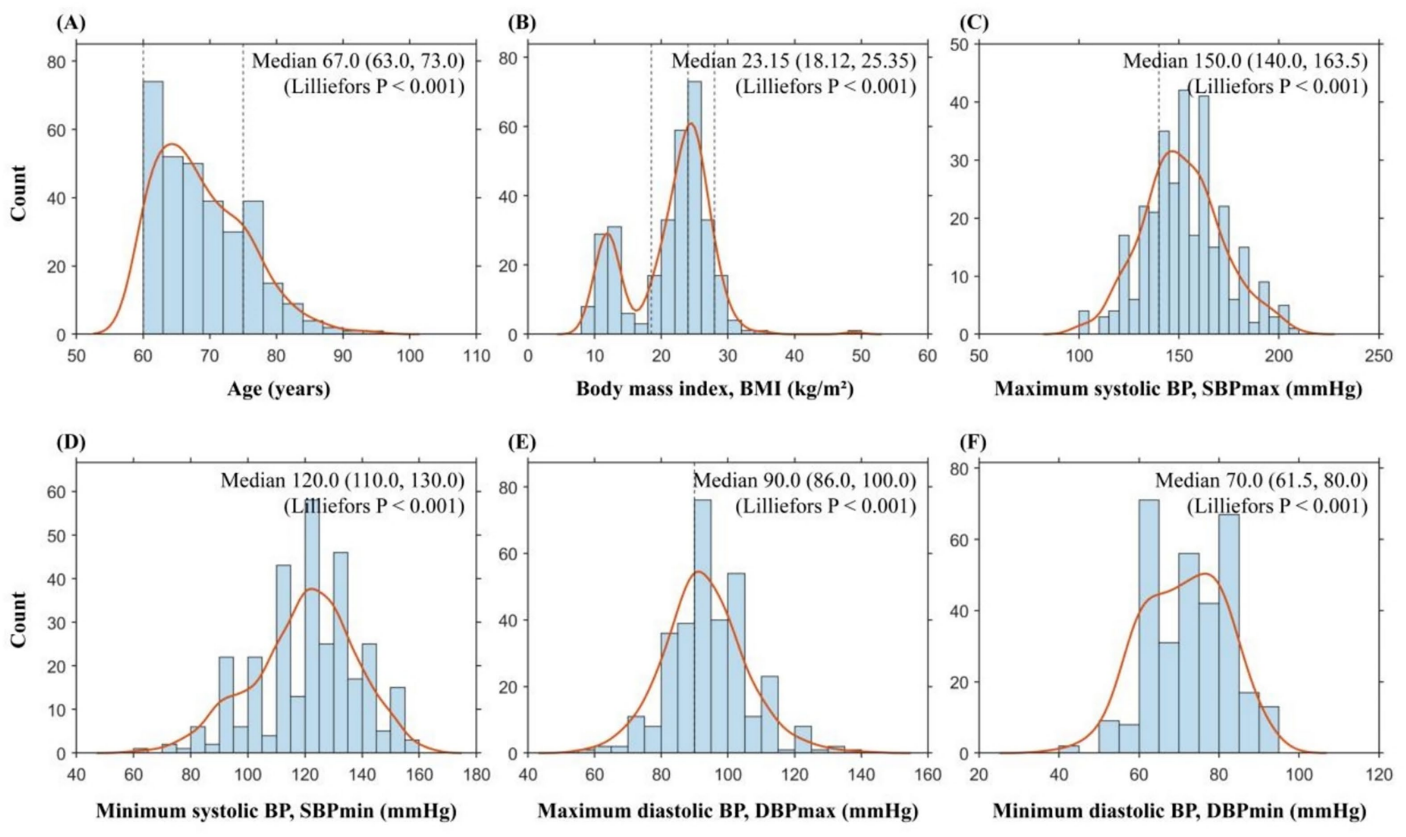
Baseline distributions of demographic, anthropometric, and blood pressure indices. Histograms with density curve overlays showing the distributions of **(A)** age, **(B)** BMI, **(C)** SBPmax, **(D)** SBPmin, **(E)** DBPmax, and **(F)** DBPmin. Values are summarized as median (IQR) within each panel. Normality was assessed using the Lilliefors test (all *p* < 0.001). BMI, body mass index; BP, blood pressure; SBP, systolic blood pressure; DBP, diastolic blood pressure; IQR, interquartile range.

A total of 408 questionnaires were collected during the study period. After excluding 34 respondents aged <60 years and 58 questionnaires with missing key demographic information, logical inconsistencies, or incomplete core items during quality control, 316 participants were included in the final analysis. The median age was 67.0 years (IQR: 63.0, 73.0), and 161 participants were female (50.9%). The median BMI was 23.15 kg/m^2^ (IQR: 18.12, 25.35). Based on participants’ self-reported recent BP fluctuation ranges, the median upper systolic BP value (SBPmax) and median upper diastolic BP value (DBPmax) were 150.0 mmHg (IQR: 140.0, 163.5) and 90.0 mmHg (IQR: 86.0, 100.0), respectively. The Lilliefors test indicated that all continuous variables were non-normally distributed (all *p* < 0.001). Baseline characteristics are summarized in Table 1 and Figure 1.

**Table 1:** Baseline characteristics of the study participants (*n* = 316).

Variable	Value, median (IQR) or *n* (%)
Age, years	67.0 (63.0, 73.0)
Female sex	161 (50.9)
BMI, kg/m^2^	23.15 (18.12, 25.35)
SBPmax, mmHg	150.0 (140.0, 163.5)
SBPmin, mmHg	120.0 (110.0, 130.0)
DBPmax, mmHg	90.0 (86.0, 100.0)
DBPmin, mmHg	70.0 (61.5, 80.0)

Continuous variables are presented as median (IQR), and categorical variables as *n* (%). Normality was assessed using the Lilliefors test; all continuous variables showed non-normal distributions (all *p* < 0.001). BMI, body mass index; BP, blood pressure; SBP, systolic blood pressure; DBP, diastolic blood pressure; IQR, interquartile range. SBPmax/SBPmin denote the self-reported upper/lower systolic BP values within the participant’s recent BP fluctuation range; DBPmax/DBPmin denote the corresponding self-reported upper/lower diastolic BP values.

### 3.2 Hypertension awareness

In terms of hypertension-related knowledge (Table 2), 241 participants (76.3%) were aware of the ideal/normal BP range, and 179 (56.6%) correctly identified the standardized BP measurement rules (the “four fixed” rule (fixed time, fixed position, fixed arm/site, and fixed device)). Awareness of behavioral risks was high, with 281 (88.9%) and 275 (87.0%) recognizing the harms of smoking and alcohol consumption, respectively.

**Table 2:** Hypertension-related knowledge among study participants (*n* = 316).

Variable	Value, *n* (%)
Know ideal/normal BP range	241 (76.3)
Knows standardized BP measurement rules (“four fixed” rule)	179 (56.6)
Knows smoking is harmful to BP	281 (88.9)
Knows alcohol consumption is harmful to BP	275 (87.0)

Data are presented as *n* (%). The “four fixed” rule refers to BP measurement performed at a fixed time, in a fixed position, using a fixed arm, and with a fixed device.

BP, blood pressure.

### 3.3 Health behaviors and management status

Self-management behaviors showed substantial gaps (Table 3). Although 238 participants (75.3%) reported having a home BP monitor, only 168 (53.2%) measured BP regularly. Overall, 236 participants (74.7%) were currently or previously using antihypertensive medications. Among the reported medication-related behaviors, the most frequently reported behavior was always following physician instructions (143, 45.3%). Among problem behaviors, the most frequently reported was stopping or reducing antihypertensive medication because BP seemed better (77, 24.4%), followed by stopping or reducing medication due to discomfort (58, 18.4%) and switching medication based on others’ advice (53, 16.8%). The distribution of the five reported medication-related behavior categories is summarized in Table 3.

**Table 3:** Hypertension self-management behaviors and management status among study participants (*n* = 316).

Variable	Value, *n* (%)
Currently/ever on antihypertensive medication	236 (74.7)
Regular BP monitoring	168 (53.2)
Home BP monitor ownership	238 (75.3)
Reported medication-related behaviors	
Always followed physician instructions	143 (45.3)
Stopped/reduced medication because BP seemed better	77 (24.4)
No hypertension/not taking antihypertensive medication	70 (22.2)
Stopped/reduced medication due to discomfort	58 (18.4)
Switched medication based on others’ advice	53 (16.8)

Data are presented as *n* (%). Regular BP monitoring indicates self-reported routine BP measurement. Medication-related behaviors were based on multiple-choice responses; therefore, percentages do not sum to 100%. Percentages were calculated using the total sample (*n* = 316) as the denominator.

BP, blood pressure.

### 3.4 Health education needs, barriers, and preferred formats

**Figure 2:**
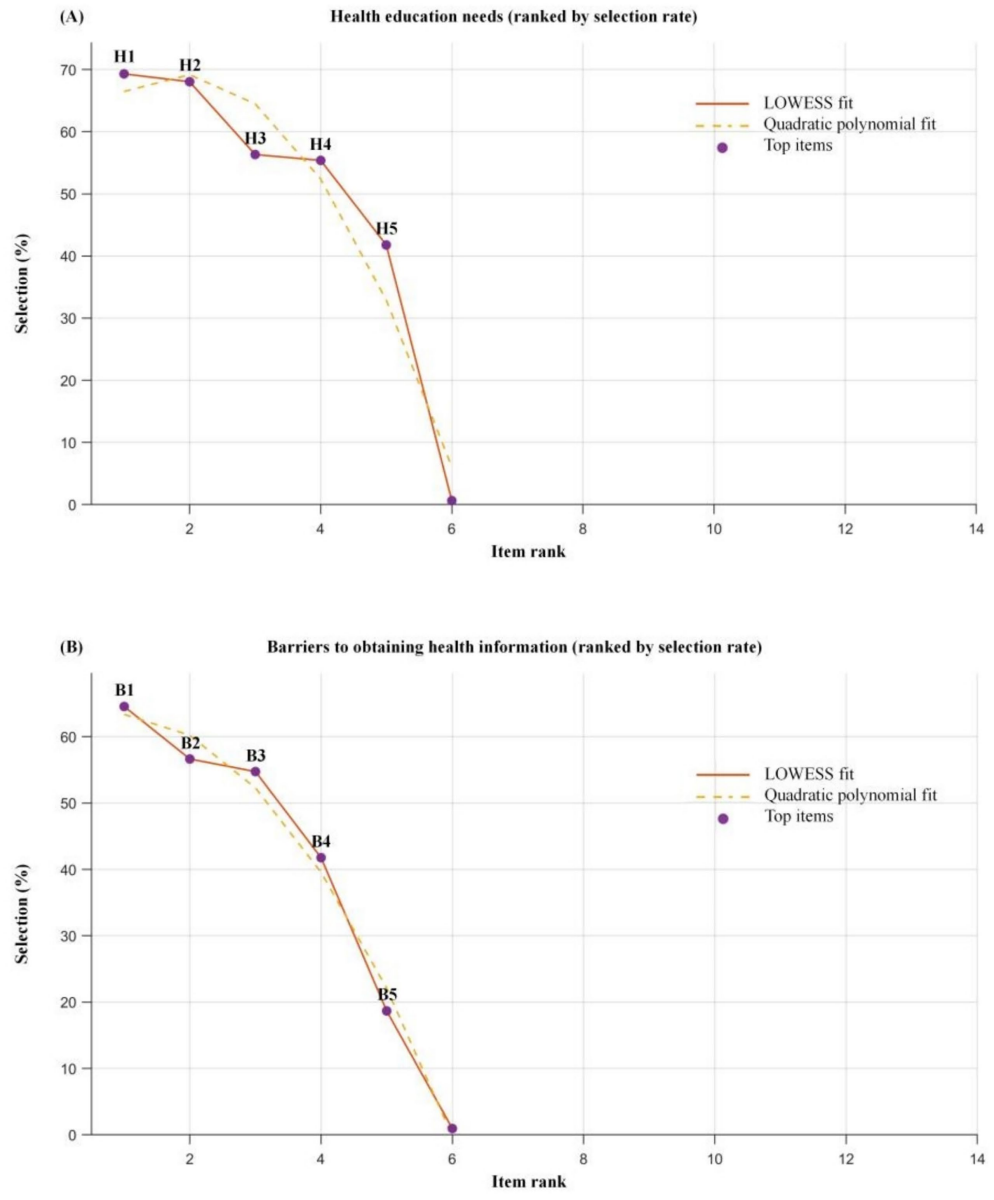
Health education needs and barriers to obtaining health information ranked by selection rate. **(A)** Health education needs ranked by selection rate. **(B)** Barriers to obtaining health information ranked by selection rate. The *y*-axis indicates the selection rate (%), and the *x*-axis indicates the item rank (sorted by selection rate). Purple markers label the top-ranked items in each domain (H1–H5 in panel **A**; B1–B5 in panel **B**). The solid line represents the LOWESS fit, and the dashed line represents the quadratic polynomial fit.

The assessment of health education needs and information barriers (Table 4; Figure 2) showed that Healthy Diet/Low-salt Recipes (219, 69.3%) and Older-adult-appropriate Exercise (215, 68.0%) were the most frequently selected education topics. The most commonly reported barrier to obtaining health information was information overload and difficulty in authenticity verification (204, 64.6%), followed by deeply technical terminology (179, 56.6%). Offline lectures/face-to-face consultation (257, 81.3%) remained the most preferred format for receiving health education.

**Table 4:** Health education needs, barriers to obtaining health information, and preferred format for hypertension management among older adults (*n* = 316).

Domain	Item (code)	Value, *n* (%)
Health education needs	Healthy Diet/Low-salt Recipes (H1)	219 (69.3)
	Older-adult-appropriate Exercise (H2)	215 (68.0)
	How to correctly measure blood pressure (H3)	178 (56.3)
	Correct Usage and Precautions for Antihypertensive Medications (H4)	175 (55.4)
	How to manage hypertensive emergencies (e.g., sudden dizziness) (H5)	132 (41.8)
Barriers to obtaining health information	Information overload and difficulty in authenticity verification (B1)	204 (64.6)
	Deeply technical terminology (B2)	179 (56.6)
	Worried that the information is false and being deceived (e.g., health product promotions) (B3)	173 (54.7)
	Do not know where to find reliable information (B4)	132 (41.8)
	No one to discuss with; forget after learning (B5)	59 (18.7)
Preferred format	Offline lectures/face-to-face consultation	257 (81.3)

Values are presented as *n* (%). Items were grouped into three domains: health education needs, barriers to obtaining health information, and preferred format. Participants could select multiple options within each domain; therefore, percentages do not sum to 100%. Item codes in Figure 2 correspond to Table 4 items (H1–H5 for health education needs; B1–B5 for barriers). Percentages were calculated using the total sample (*n* = 316) as the denominator.

### 3.5 Factors associated with regular BP monitoring

**Figure 3:**
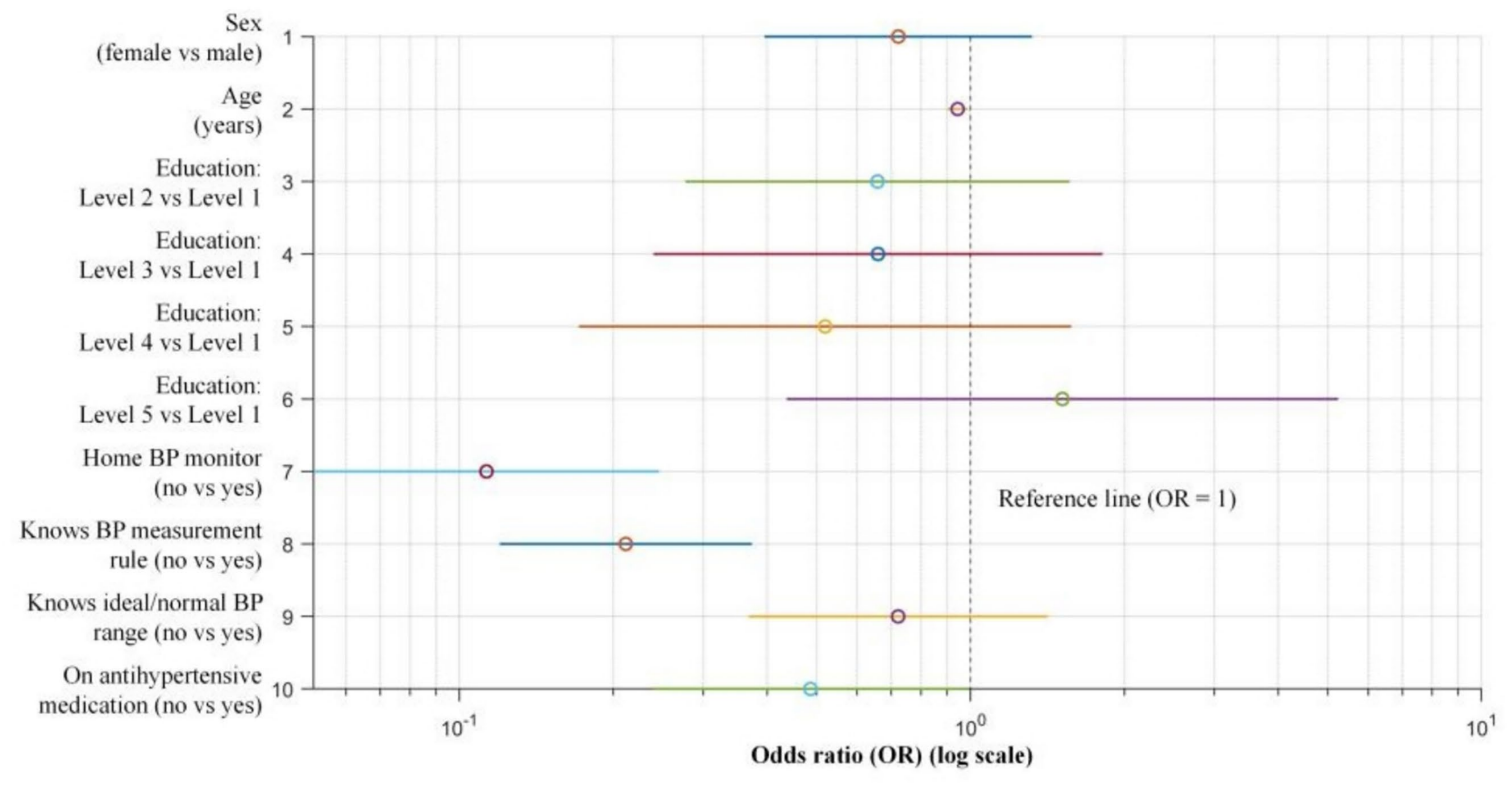
Forest plot of multivariable logistic regression for factors associated with regular blood pressure monitoring. Points indicate odds ratios (ORs) and horizontal lines indicate 95% confidence intervals (CIs) on a logarithmic scale. The vertical dashed line represents the null effect (OR = 1). The dependent variable was regular blood pressure (BP) monitoring (1 = yes, 2 = no). Education was modeled as a categorical variable with Level 1 (primary school or below) as the reference group; comparisons are Level 2–Level 5 vs. Level 1 (Level 2 = junior high school; Level 3 = senior high school/secondary technical school; Level 4 = junior college; Level 5 = bachelor’s degree or above). Binary predictors are presented as no vs. yes, including home BP monitor ownership, awareness of standardized BP measurement rules (“fixed time, fixed posture, fixed arm/site, and fixed device”), awareness of the ideal/normal BP range, and current or prior use of antihypertensive medication.

Multivariable logistic regression was performed to identify factors associated with regular BP monitoring (Table 5; Figure 3). Older age was independently associated with lower odds of regular monitoring (OR = 0.944 per year; 95% CI: 0.901–0.988; *p* = 0.013). Participants without a home BP monitor (no vs. yes) had markedly lower odds of regular monitoring (OR = 0.113; 95% CI: 0.052–0.246; *p* < 0.001), and those who did not know the standardized BP measurement rules (no vs. yes; “four fixed” rule) were also less likely to monitor regularly (OR = 0.212; 95% CI: 0.120–0.373; p < 0.001). In addition, participants not currently or previously using antihypertensive medication (no vs. yes) showed lower odds of regular monitoring (OR = 0.486; 95% CI: 0.240–0.986; *p* = 0.046). Sex, educational level, and awareness of the ideal/normal BP range were not significantly associated with regular BP monitoring (all *p* > 0.05).

**Table 5:** Multivariable logistic regression analysis of factors associated with regular blood pressure monitoring.

Predictor	Odds ratio (OR)	95% CI	*p*-value
Sex (Female vs. Male)	0.722	0.395–1.320	0.290
Age (per year)	0.944	0.901–0.988	0.013
Education (Level 2 vs. Level 1)	0.658	0.277–1.563	0.343
Education (Level 3 vs. Level 1)	0.659	0.240–1.812	0.419
Education (Level 4 vs. Level 1)	0.519	0.171–1.575	0.245
Education (Level 5 vs. Level 1)	1.513	0.437–5.239	0.514
Home BP monitor (no vs. yes)	0.113	0.052–0.246	< 0.001
Knows BP measurement rule (no vs. yes)	0.212	0.120–0.373	< 0.001
Knows ideal/normal BP range (no vs. yes)	0.722	0.368–1.416	0.343
On antihypertensive medication (no vs. yes)	0.486	0.240–0.986	0.046

The dependent variable was regular blood pressure (BP) monitoring (1 = yes, 2 = no). Results are presented as odds ratios (ORs) with 95% confidence intervals (CIs) and *p* values. Sex was coded as female vs male, and age was modeled as a continuous variable (years). Education was modeled as a categorical variable with Level 1 (primary school or below) as the reference group; comparisons were Level 2–Level 5 vs Level 1 (Level 2 = junior high school; Level 3 = senior high school/secondary technical school; Level 4 = junior college; Level 5 = bachelor’s degree or above). Home BP monitor, knows BP measurement rule, knows ideal/normal BP range, and on antihypertensive medication were coded as yes/no, and ORs are presented as no vs yes to match the forest plot labeling.

### 3.6 Clustering-derived profiles and heterogeneous outcomes

**Figure 4:**
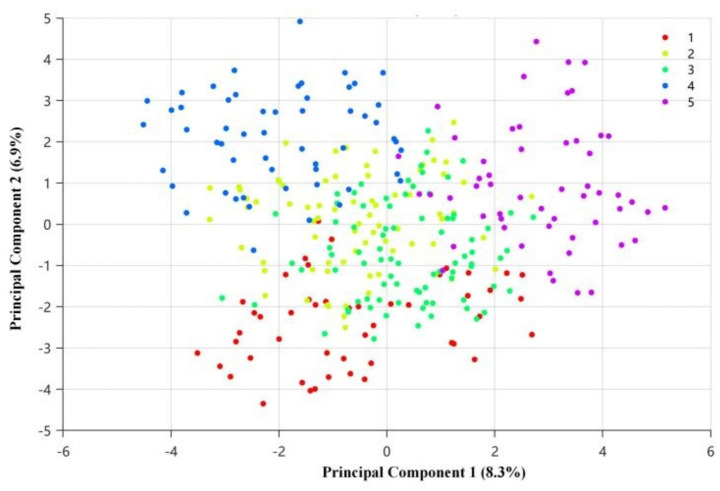
Clustering-derived management profiles visualized by principal component analysis (k = 5). Scatter plot of participants projected onto the first two principal components derived from the clustering features. PC1 explains 8.3% and PC2 explains 6.9% of the total variance. Colors indicate cluster membership (Cluster 1–5; labeled as 1–5 in the legend) identified by k-means clustering (k = 5). Each dot represents one participant.

**Figure 5:**
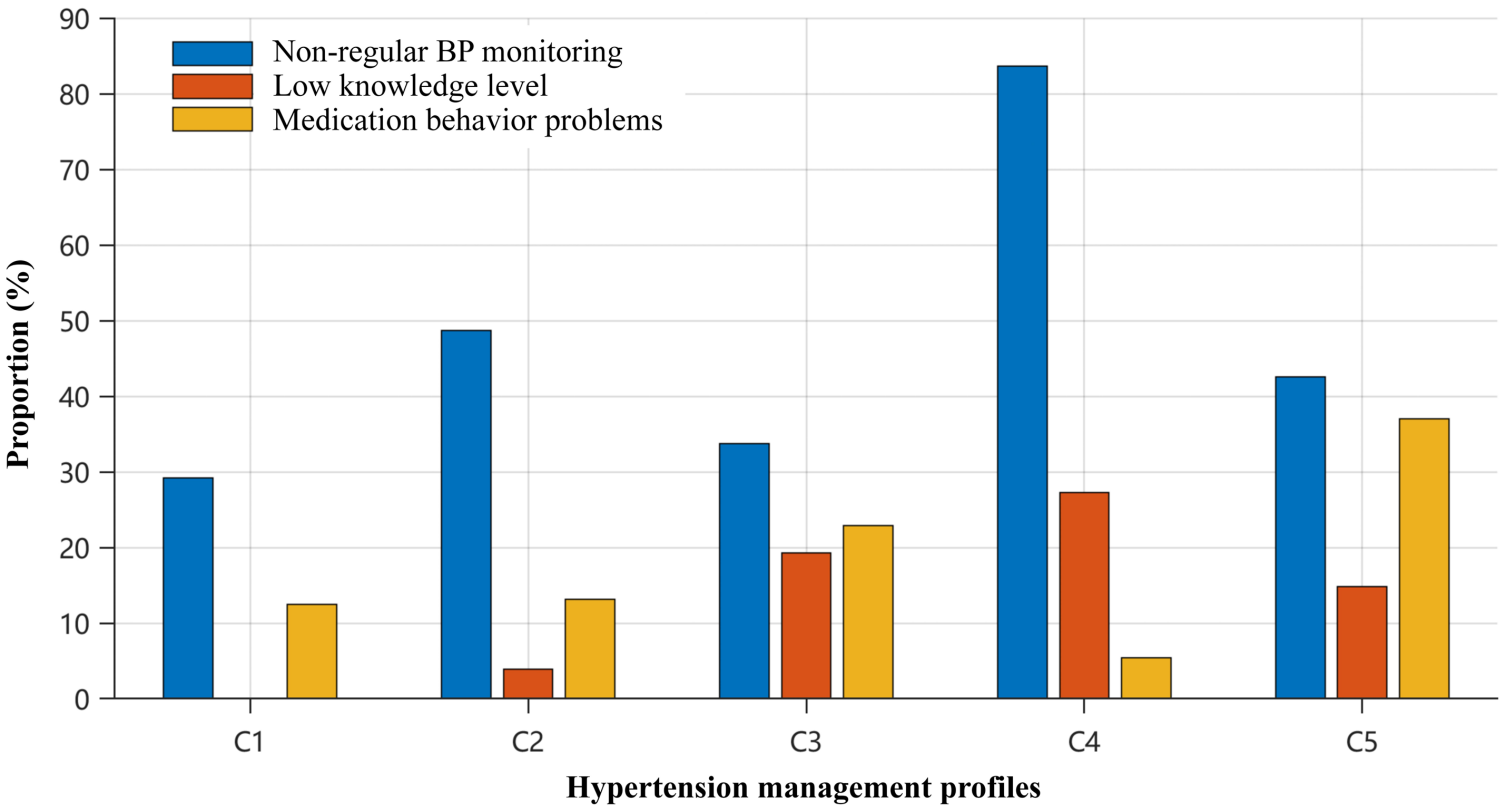
Comparison of key outcome indicators across clusters (C1–C5). Proportions of three key outcomes across the five clusters (C1–C5): insufficient/non-regular blood pressure (BP) monitoring, low knowledge level, and medication behavior problems. Bars represent the percentage of participants within each cluster exhibiting each outcome. C1–C5 correspond to Cluster 1–5 in Table 6. BP, blood pressure.

Beyond the regression analysis of regular BP monitoring, k-means clustering identified five distinct hypertension management profiles. In the PCA visualization, the first two principal components explained 15.2% of the total variance (PC1: 8.3%; PC2: 6.9%) (Figure 4). Significant heterogeneity was observed across clusters in key outcome indicators after FDR adjustment (all P_FDR < 0.001; Table 6), including age, non-regular BP monitoring, low knowledge level, and medication behavior problems (Figure 5). Specifically, Cluster 1 (*n* = 48, 15.2%) represented the High-knowledge/regular-monitoring profile, with the lowest non-regular BP monitoring rate (29.2%) and 0.0% prevalence of low knowledge level. Cluster 4 (*n* = 55, 17.4%) reflected the Low-monitoring/low-resource profile, characterized primarily by a very high non-regular BP monitoring rate (83.6%) and a high prevalence of low knowledge level (27.3%), rather than by the highest level of medication-related problems. In contrast, Cluster 5 (*n* = 54, 17.1%) corresponded to the Medication-problem profile, characterized by the highest prevalence of medication behavior problems (37.0%) despite only moderate non-regular BP monitoring rate (42.6%). The remaining profiles showed intermediate patterns: Cluster 2 (*n* = 76, 24.1%) represented the Intermediate-management/higher-BMI profile with a non-regular BP monitoring rate of 48.7%, whereas Cluster 3 (*n* = 83, 26.3%) represented the Knowledge-deficit/medication-problem profile, with elevated prevalence of low knowledge level (19.3%) and medication behavior problems (22.9%).

**Table 6:** Key outcome indicators across the five hypertension management profiles (k-means clustering, k = 5).

Outcome Measure	Cluster 1 (*n* = 48)	Cluster 2 (*n* = 76)	Cluster 3 (*n* = 83)	Cluster 4 (*n* = 55)	Cluster 5 (*n* = 54)	P_FDR
Group Description	High-knowledge/regular-monitoring profile	Intermediate-management/higher-BMI profile	Knowledge-deficit/medication-problem profile	Low-monitoring/low-resource profile	Medication-problem profile	–
Mean Age (years)	68.3	69.2	71.5	64.5	68.2	< 0.001
Non-regular BP monitoring (%)	29.2%	48.7%	33.7%	83.6%	42.6%	< 0.001
Low Knowledge Level (%)	0.0%	4.0%	19.3%	27.3%	14.8%	< 0.001
Medication Behavior Problems (%)	12.5%	13.2%	22.9%	5.5%	37.0%	< 0.001

Values are presented as mean (age) or percentage (categorical outcomes). non-regular BP monitoring indicates the proportion of participants who did not measure blood pressure regularly. Low knowledge level indicates the proportion classified as having lower hypertension-related knowledge based on the four-item knowledge score, grouped using the median split method. Scores of 0–2 were classified as low knowledge level, whereas scores of 3–4 were classified as high knowledge level. Medication behavior problems indicates the proportion reporting medication non-adherence behaviors (defined in Table 3). P_FDR denotes false discovery rate–adjusted *p* values for between-cluster comparisons (Kruskal–Wallis test for age; chi-square tests for categorical outcomes). All comparisons remained significant after FDR adjustment (all P_FDR < 0.001).

## 4 Discussion

In this cross-sectional survey of Chinese adults aged 60 years and older recruited from community-based and outpatient clinical settings during January 2026, we provide an integrated characterization of hypertension-related knowledge, self-management behaviors, and educational needs, complemented by multivariable modeling and unsupervised profiling (25). Three main findings emerged that collectively highlight the importance of translating guideline-consistent self-management recommendations into older-adult–friendly, actionable routines (26). First, although awareness of several hypertension-related concepts and behavioral risks was relatively high, substantial gaps persisted in actionable self-management behaviors—most notably regular BP monitoring, whose correct implementation and consistent use are central to out-of-office BP management (26). Second, education needs were strongly oriented toward practical lifestyle guidance (healthy diet/low-salt recipes and older-adult-appropriate exercise), while major barriers centered on information overload, difficulty verifying authenticity, and overly technical terminology, which is consistent with growing evidence on limited digital health literacy and misinformation vulnerability among older adults (27, 28). Third, unsupervised clustering revealed meaningful heterogeneity in key outcomes (all P_FDR < 0.001), consistent with prior work showing that engagement and self-management behaviors can cluster into distinct profiles that may require different support strategies (29). Importantly, the regression analysis and clustering analysis addressed different but complementary questions: the former focused on determinants of one key actionable behavior (regular BP monitoring), whereas the latter characterized broader heterogeneity in management patterns and educational needs. Collectively, these results suggest that health education for Chinese adults aged 60 years and older in similar community-based and outpatient care settings may benefit from moving beyond one-size-fits-all messaging toward profile-guided, barrier-aware intervention design (28, 29).

A striking observation in this sample was a partial disconnect between knowledge indicators and actual self-management behavior, a pattern that has also been reported in older hypertensive populations where monitoring adherence is often substantially lower than medication adherence (30, 31). While a considerable proportion of participants reported knowing the ideal/normal BP range and recognizing the harms of smoking and alcohol consumption, only 53.2% reported regular BP monitoring, despite 75.3% of households having a home BP monitor; similarly, population-based evidence in middle-to-older adults has shown that ownership or access does not necessarily translate into regular self-measured BP monitoring (32). This gap is clinically important because out-of-office BP monitoring (including HBPM) is recommended to improve diagnostic accuracy (e.g., masked/white-coat hypertension) and to support ongoing evaluation and treatment adjustment, and its value depends on regular measurement using standardized procedures (33). In multivariable logistic regression, older age was independently associated with lower odds of regular monitoring, consistent with reports that monitoring adherence in older adults is shaped by functional burden, health-service engagement, and education exposure rather than awareness alone (31). More importantly, lack of a home BP monitor and lack of awareness of standardized BP measurement rules (“four fixed”) showed strong negative associations with regular monitoring (both *p* < 0.001), whereas awareness of the ideal/normal BP range was not independently associated—supporting the interpretation that, in older cohorts, device access and procedural know-how (how to measure correctly and consistently) are more proximal drivers of monitoring behavior than general conceptual knowledge (30, 33, 34). In addition, participants not currently/ever using antihypertensive medication were less likely to monitor regularly, aligning with evidence that home BP monitoring is closely linked to broader treatment engagement and adherence-related behaviors (31). However, this regression model primarily captured operational determinants of BP monitoring and did not include broader lifestyle, barrier-related, or psychosocial variables discussed in the Introduction; therefore, its explanatory scope should be interpreted as behavior-specific rather than comprehensive.

The education-needs profile observed in this sample of Chinese adults aged 60 years and older offers direct implications for intervention content and delivery in similar settings. In our sample, the highest-demand topics—healthy diet/low-salt recipes and older-adult-appropriate exercise—mirror guideline priorities that emphasize sodium reduction and regular physical activity as core nonpharmacologic strategies for hypertension management (35). In parallel, information overload and difficulty verifying authenticity emerged as the most frequently reported barriers, consistent with evidence that older adults face substantial challenges navigating high-volume, mixed-quality health information and judging credibility in digital environments (36). These barriers can be amplified by age-related sensory/cognitive changes and variable health/digital literacy, making it harder to extract actionable steps from fragmented or overly technical content (36). Accordingly, educational materials may benefit from prioritizing plain language, standardized key messages, and task-oriented tools (e.g., step-by-step checklists for BP measurement, common pitfalls, and troubleshooting), because simplifying presentation improves comprehension and usability compared with standard-language formats (37). Notably, offline lectures/face-to-face consultation remained the most preferred format in our survey, supporting a delivery strategy centered on clinician- or institution-endorsed, interactive education—particularly for similar populations who value trusted sources and opportunities for clarification (36).

Importantly, the clustering results in this study indicate that different dimensions of hypertension self-management do not necessarily co-vary uniformly across subgroups. Rather than representing a single continuum of overall engagement, the identified profiles reflect dimension-specific heterogeneity across monitoring behavior, knowledge level, and medication-related problems. The clustering results extend these insights by demonstrating clinically relevant heterogeneity in hypertension self-management that can be obscured by aggregate statistics, consistent with recent work showing distinct engagement/monitoring patterns in hypertension self-management and home BP measurement programs (38–40). Cluster 1 represented a High-knowledge/regular-monitoring profile (non-regular BP monitoring 29.2%; low knowledge 0.0%), suggesting a subgroup already broadly aligned with recommended management behaviors (41). In contrast, Cluster 4 reflected a Low-monitoring/low-resource profile with the highest non-regular BP monitoring (83.6%) and the highest low-knowledge prevalence (27.3%), but not the highest level of medication-related problems, identifying a priority target for intensified, highly practical support focused on reducing operational burden—such as simplifying routines, providing hands-on skills training for standardized measurement, and strengthening external support through family/caregiver involvement or community-based assistance (41, 42). Cluster 5 corresponded to a Medication-problem profile (medication behavior problems 37.0%) despite only moderate non-regular monitoring (42.6%), implying that adherence barriers may persist even when monitoring is not severely impaired; this aligns with evidence that intentional non-adherence in older adults with hypertension is often driven by medication beliefs, side-effect concerns, and deliberate dose modification (43, 44). Cluster 3 showed a Knowledge-deficit/medication-problem profile (low knowledge 19.3%; medication behavior problems 22.9%), suggesting a need for combined strategies that reinforce “how-to” knowledge while directly addressing adherence barriers via counseling and practical medication-management supports (21, 43). Cluster 2 displayed an intermediate pattern and was characterized as an Intermediate-management/higher-BMI profile, reinforcing that lifestyle-oriented education remains relevant even when knowledge deficits are not prominent (21, 41). Taken together, these profiles support a tailored approach: technique- and routine-focused interventions for groups with monitoring-related deficits, trusted-source navigation support to mitigate information-quality barriers, and belief−/side-effect–oriented counseling plus regimen simplification for medication-problem groups (43, 44). In practice, a brief screening tool based on a small set of discriminative features (e.g., home monitor ownership, awareness of standardized measurement rules, and key non-adherence behaviors) could help triage individuals into appropriate education pathways, consistent with calls to integrate feasible adherence assessment and targeted support into routine hypertension care (43, 44).

## 5 Limitations and future directions

Several limitations should be acknowledged. First, the cross-sectional design precludes causal inference; observed associations may reflect unmeasured confounding or reverse causality. Second, key behaviors and knowledge were self-reported, which may introduce recall and social desirability bias. Third, the multivariable regression model focused on proximal determinants of regular BP monitoring and did not include broader lifestyle, perceived barrier, or psychosocial/contextual variables. As a result, the model explains one key self-management behavior rather than the full multidimensional complexity of hypertension self-management. Fourth, clustering solutions depend on feature selection, scaling, and algorithmic settings; although the five-cluster solution yielded interpretable profiles and significant between-cluster differences after FDR adjustment, external validation in independent cohorts is warranted.

In addition, the questionnaire was specifically developed for this study and primarily used as an item-based survey instrument. Although its content validity and face validity were strengthened through multidisciplinary expert review and pilot testing in older adults before formal administration, it did not undergo full psychometric reliability testing and was not a fully psychometrically validated scale, which may limit comparability with studies using standardized instruments. Finally, the survey was conducted in communities surrounding a single rehabilitation hospital in Shanghai and in related outpatient clinical settings; therefore, the findings should be interpreted as applying primarily to Chinese adults aged 60 years and older in similar community-based and outpatient care contexts, and generalizability to other regions, healthcare systems, or broader populations of older adults may be limited (38, 43).

Despite these limitations, our findings align with contemporary hypertension guidance emphasizing that self-measured BP monitoring, when embedded in structured support and follow-up, can strengthen self-management and improve BP control in real-world settings (45). Accordingly, hypertension education for similar populations should prioritize actionable lifestyle modules and translate recommendations into plain-language, stepwise tasks that are feasible in daily routines and adaptable to functional limitations (46). Because patient-facing online materials for home BP monitoring are often written above recommended readability levels and show variability in evidence alignment, providing clinician-endorsed, vetted resources and standardized checklists may reduce cognitive burden and improve usability for older adults (47). In parallel, older adults’ ability to navigate digital health information and appraise credibility is heterogeneous; therefore, interventions should explicitly address information overload and authenticity verification by strengthening credibility-assessment strategies and directing users to trusted channels (48). Finally, evidence from tailored/self-management intervention trials supports moving beyond uniform materials toward targeted pathways (e.g., device-access/skills support for low-monitoring/low-resource profiles and behavior-focused adherence support for medication-problem profiles), which warrants prospective evaluation for sustained effects on monitoring, adherence, BP control, and clinical outcomes (49).

## 6 Conclusions and recommendations

In this cross-sectional survey of Chinese adults aged 60 years and older recruited from community-based and outpatient clinical settings, substantial gaps were observed between hypertension-related knowledge and the adoption of regular self-management behaviors, particularly BP monitoring. Although awareness of several hypertension-related concepts was relatively high, regular BP monitoring remained suboptimal, and practical barriers to obtaining and using health information were common. In addition, the profile-based analysis showed that different dimensions of hypertension self-management did not align uniformly across subgroups, highlighting meaningful heterogeneity in monitoring behavior, knowledge level, and medication-related problems.

These findings suggest that hypertension education in similar populations and care settings should move beyond uniform messaging and prioritize profile-informed, barrier-aware strategies. Practical recommendations include strengthening access to home BP monitoring, improving procedural knowledge through plain-language and stepwise instruction, addressing information overload and credibility concerns, and tailoring support according to dominant management barriers, such as monitoring-related deficits or medication-related problems. Future studies should evaluate whether such targeted approaches can improve regular BP monitoring, treatment adherence, BP control, and longer-term clinical outcomes.

## Data Availability

The anonymized data supporting the findings of this study are available from the corresponding author upon reasonable request and may be shared in accordance with institutional and ethical guidelines.
