# Supplementary material for: Hypertension management profiles in Chinese adults aged 60 years and older
Source: Front Public Health. 2026 May 19;14:1807538. doi: 10.3389/fpubh.2026.1807538 (PMC13226592; doi:10.3389/fpubh.2026.1807538)
Supplement: Supplementary file 1 [file Table_1.docx]

**Survey Questionnaire on Behavioral Cognition and Needs of Hypertension in the Elderly Population**

**Questionnaire number: ________**

**Date of survey: ____ year ____ month ____ day**

**Questionnaire Instructions: Dear Sir or Madam, we are conducting a survey on hypertension prevention and management among middle-aged and elderly individuals to understand the knowledge, behaviors, and health education needs related to hypertension in this population. This questionnaire is designed for anonymous completion, and all data will be used solely for statistical analysis. Your personal information will be strictly confidential. Please feel free to complete the questionnaire based on your actual circumstances. We appreciate your support and participation.**

## 1. What is your gender?

## □ Male □ Female

## 2. Your age is ________ years, height is ________ cm, and weight is ________ kg.

**3. Your highest educational level:**

□ Primary school or below □ Junior high school □ Secondary vocational school or high school □ College degree □ Bachelor's degree or above

## 4. Recent blood pressure fluctuation range:

## Systolic blood pressure (diastolic pressure) ________～________ mmHg;

## Diastolic blood pressure (systolic blood pressure) ________～________ mmHg.

**5. Are you aware that the ideal blood pressure range is systolic blood pressure <120 mmHg and diastolic blood pressure <80 mmHg, while the normal blood pressure range is systolic blood pressure 90–139 mmHg and diastolic blood pressure 60–89 mmHg?**

□ Yes □ No

**6. Do you have any chronic diseases diagnosed by a physician (e.g., hypertension, diabetes mellitus, heart disease, arthritis, etc.)? (Multiple selections allowed)**

□ Hypertension □ Coronary heart disease □ Diabetes mellitus □ Stroke □ Chronic obstructive pulmonary disease □ Other diseases □ None

7. Do you actively engage in or learn about health-related knowledge on a regular basis?

□ Regularly proactively monitor and learn □ Occasionally review when encountered □ Rarely monitor unless experiencing physical discomfort □ Never monitor

**8. What knowledge about hypertension would you most like to learn? (Multiple choices allowed)**

□ How to correctly measure blood pressure

□ How to Eat Healthily (Low-Salt Diet Recipes)

□ Exercise modalities suitable for the elderly

□ Correct Usage and Precautions of Antihypertensive Drugs

□ How to manage hypertensive emergencies (e.g., sudden dizziness)

□ Others (please specify):

**9. What are your difficulties or confusions when acquiring health knowledge? (Multiple choices allowed)**

□ Too much and too mixed information, unable to determine which is correct

□ Many technical terms are incomprehensible and overly complex

□ Concerned that the information is false and falling victim to fraud (e.g., health product sales)

□ Uncertain where to find reliable information

No one discusses together, so what you learn is forgotten

□ Others (please specify):

**10. What form of health science popularization do you prefer?**

□ TV or short video (with visuals and narration, intuitive and easy to understand)

□ Illustrated brochures or articles (can be read slowly)

□ Listen to radio or health audio (e.g., podcasts)

□ Attend offline health lectures or free medical consultations (allowing face-to-face questioning with physicians)

□ Someone accompanies the activity (e.g., teaching health exercises or nutritious meals)

□ Others (please specify):

**11. How many years have you had hypertension?**

□ No hypertension □ 1–5 years □ 6–10 years □ Over 10 years

**12. Have you ever taken antihypertensive drugs or are you currently taking antihypertensive drugs?**

□ Yes □ No

**13. Which of the following conditions occurred while you were taking antihypertensive medications? (Multiple selections allowed)**

□ Discomfort occurs, leading to self-discontinuation or dose reduction

□ Discontinuing or reducing medication on one's own after perceiving improvement in blood pressure

□ Heard others recommend other medications and switched treatments

□ Always followed the doctor's advice and took medication on time and in the prescribed dosage

□ No hypertension, no antihypertensive medication

**14. What is your usual salt intake habit?**

□ Consuming excessive salt

□ Moderately salty or mild

□ Maintain a light diet

□ Minimal salt intake

**15. What is your average daily exercise duration, such as walking, brisk walking, jogging, swimming, etc.?**

□ Almost no exercise

□ Within 10 minutes

□ 10-30 minutes

□ More than 30 minutes

**16. What do you consider to be the key to controlling hypertension?**

□ Take antihypertensive medications on time

□ low salt diet

□ exercise training

□ Maintain a positive mindset

**17. Are you aware of the harm smoking causes to blood pressure?**

□ Knows □ Don't know

**18. How many years have you been smoking in total?**

□ Never smoked

□ 1-10 years

□ 11-20 years

□ 21 years or older

**19. Are you aware of the hazards of alcohol consumption on blood pressure?**

□ Knows □ Does not know

**20. How many years have you been drinking alcohol in total?**

□ Never drink alcohol

□ 1-10 years

□ 11-20 years and above

□ 21 years or older

**21. Do you measure your blood pressure on time? If yes, how often?**

□ Yes □ No

**22. Do you have a sphygmomanometer at home?**

□ Yes □ No

**23. Are you aware that blood pressure measurement requires "fixed time, fixed position, fixed site, and fixed sphygmomanometer"?**

□ Knows □ Don't know

**24. Do you regularly attend follow-up visits to the hospital or consult with physicians?**

□ Yes, follow-up visits every 1-3 months

□ Yes, follow-up every six months

□ Rarely attended follow-up visits, only going for medication dispensing

□ Never followed up for re-examination

**25. Which of the following symptoms may occur with elevated blood pressure? (Multiple choices allowed)**

□ dizzy

□ feeling of fullness in the head

□ headache

□ tinnitus

□ sense of suppression in the chest

□ Palpitations

□ Blackouts

□ Blurred vision

**老年群体高血压行为认知与需求调查问卷**

**问卷编号：** ________

**调查日期：** ____年____月____日

**问卷说明：尊敬的先生、女士，您好！我们正进行一项关于中老年人高血压防治的调查，旨在了解老年人群对高血压相关知识、行为及健康教育需求的情况，本问卷实行匿名填写，所有数据仅用于统计分析，我们将对您的个人信息严格保密，请您依据实际情况放心填写，感谢您的支持与参与！**

## ****1.您的性别是？****

## □ 男 □ 女

## ****2.您的年龄****________岁****，身高**** ________cm，****体重**** ________kg

**3.您的最高文化程度：**

□ 小学或以下 □初中 □ 中专或高中 □ 大专 □本科及以上

## ****4.近期您的血压波动范围：****

## **收缩压（上压）**________～________mmHg；

## **舒张压（下压）________～________mmHg。**

**5.您是否了解理想血压的范围是收缩压＜120mmHg、舒张压＜80mmHg，正常血压的范围是收缩压90~139mmHg，舒张压60~89mmHg？**

□ 是 □ 否

**6.您是否患有经医生诊断的慢性疾病（如高血压、糖尿病、心脏病、关节炎等）？（可多选）**

□高血压 □ 冠心病 □ 糖尿病 □ 脑中风 □ 慢性阻塞性肺病 □其他疾病 □ 无

**7.您平时会主动关注或学习健康方面的知识吗**？

□ 经常主动关注和学习 □ 偶尔看到会了解一下 □很少关注，除非身体不舒服 □从不关注

**8.您最想了解关于高血压的哪些知识？（可多选）**

□ 如何正确测量血压

□ 怎么吃才健康（低盐食谱）

□ 适合老年人的运动方式

□ 降压药的正确用法和注意事项

□ 高血压急症（如突然头晕）如何处理

□ 其他（请注明）：

**9.在获取健康知识时，您的困难或困惑是什么？（可多选）**

□ 信息太多太杂，不知道哪个是对的

□ 很多专业术语看不懂，太深奥

□ 担心信息是假的，上当受骗（比如保健品推销）

□ 不知道去哪里找靠谱的信息

□ 没人一起讨论，学了就忘

□ 其他（请注明）：

**10.您喜欢哪种形式的健康科普？**

□ 电视或短视频（有画面、有讲解，直观易懂）

□ 图文并茂的宣传册或文章（可以慢慢看）

□ 收听广播或健康音频（比如播客）

□ 参加线下的健康讲座或义诊（可以和医生面对面问）

□ 有人带着一起做（比如教做健康操、营养餐）

□ 其他（请注明）：

**11.您的高血压有多少年了？**

□ 没有高血压 □1–5年 □ 6–10年 □10年以上

**12.您是否服用过降压药或者正在服用降压药？**

□ 是 □ 否

**13.您吃降压药的时候，有出现下列哪些情况？（可多选）**

□ 感觉不舒服，自己停药或减量

□ 觉得血压好了，自己把药停了或减了

□ 听别人说其他药好，自己换过药

□ 一直听医生的，按时按量吃

□ 没有高血压，不吃降压药

**14.您平时吃盐的习惯？**

□ 吃得很咸

□ 咸淡适中

□ 吃得清淡

□ 几乎不吃盐

**15.您平均每天的运动时间，比如散步、快走、慢跑、游泳等运动？**

□ 几乎不运动

□ 10分钟以内

□ 10-30分钟

□ 30分钟以上

**16.您认为控制高血压的关键是？**

□ 按时服用降压药

□ 低盐饮食

□ 运动锻炼

□ 保持良好心态

**17.您知道吸烟对血压的危害吗？**

□知道 □ 不知道

**18.您总共吸烟多少年？**

□ 从来不吸烟

□ 1-10年

□ 11-20年

□ 21年及以上

**19.您知道饮酒对血压的危害吗？**

□ 知道 □ 不知道

**20.您总共饮酒多少年？**

□ 从来不喝酒

□ 1-10年

□ 11-20年及以上

□ 21年及以上

**21.您会按时测量血压吗？如果有是多久一次**

□ 会 □ 不会

**22.您家中有血压计吗？**

□有 □ 没有

**23.您是否知道测量血压时需要“定时间、定体位、定部位、定血压计”？**

□知道 □ 不知道

**24.您是否定期去医院复诊或询问医生？**

□ 是，每1-3个月复诊一次

□ 是，半年复诊一次

□ 很少去复诊，只有配药的时候去

□ 从不复诊

**25.您认为血压偏高可能会出现以下哪项症状？（可多选）**

□ **头晕**

□ **头胀**

□ **头痛**

□ **耳鸣**

□ **胸闷**

□ **心悸**

□ **眼前发黑**

□ **视物模糊**
